# Supplementary material for: The neural correlates of continuous feedback processing
Source: Psychophysiology. 2023 Jul 24;60(12):e14399. doi: 10.1111/psyp.14399 (PMC10851313; doi:10.1111/psyp.14399)
Supplement: Supplementary file 1 — Data S1 [file PSYP-60-e14399-s001.pdf]

Supplementary Material

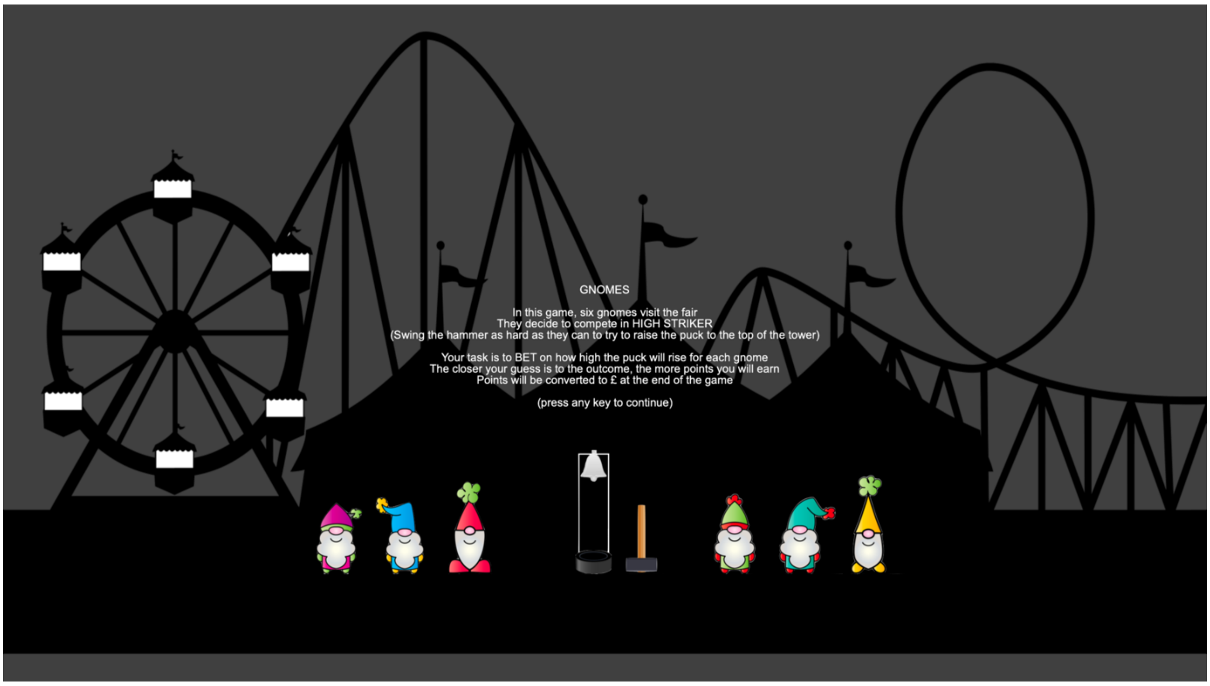

Supplementary Figure S1. First instruction screen.

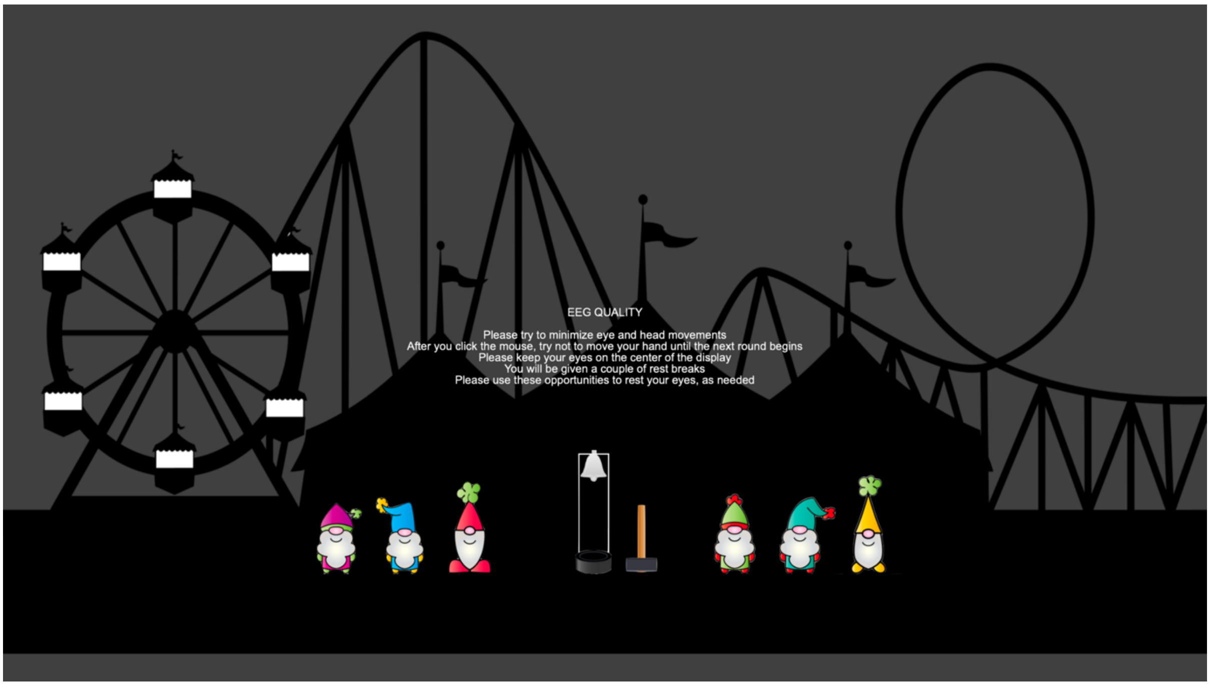

Supplementary Figure S2. Second instruction screen.

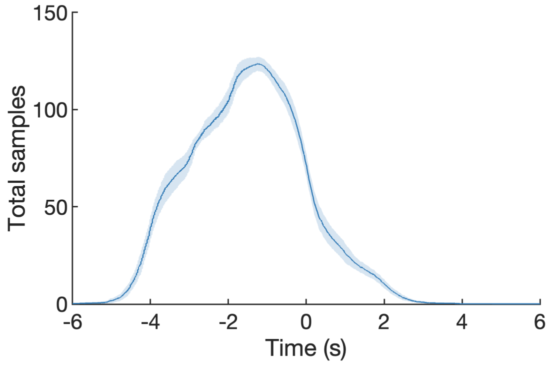

Supplementary Figure S3. Mean total number of modelled timepoints relative to the maximum reward value at 0 s. The shaded region indicates the 95% confidence interval.

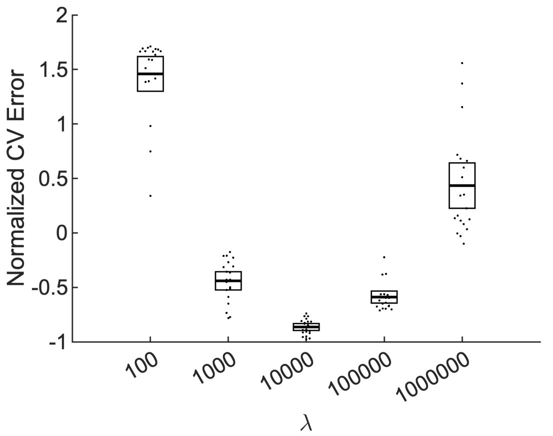

Supplementary Figure S4. Normalized cross-validation error. Dots represent individual participants, and error bars represent 95% confidence intervals. For all participants, the error was minimized by a lambda value of 10,000.

Supplementary Table 1

Mean Distance Between Response and Outcome as a Proportion of Bar Height

Outcome Probability	Mean (Proportion)	95% CI
High	0.10	[0.07, 0.12]
Medium	0.12	[0.10, 0.13]
Low	0.15	[0.13, 0.16]

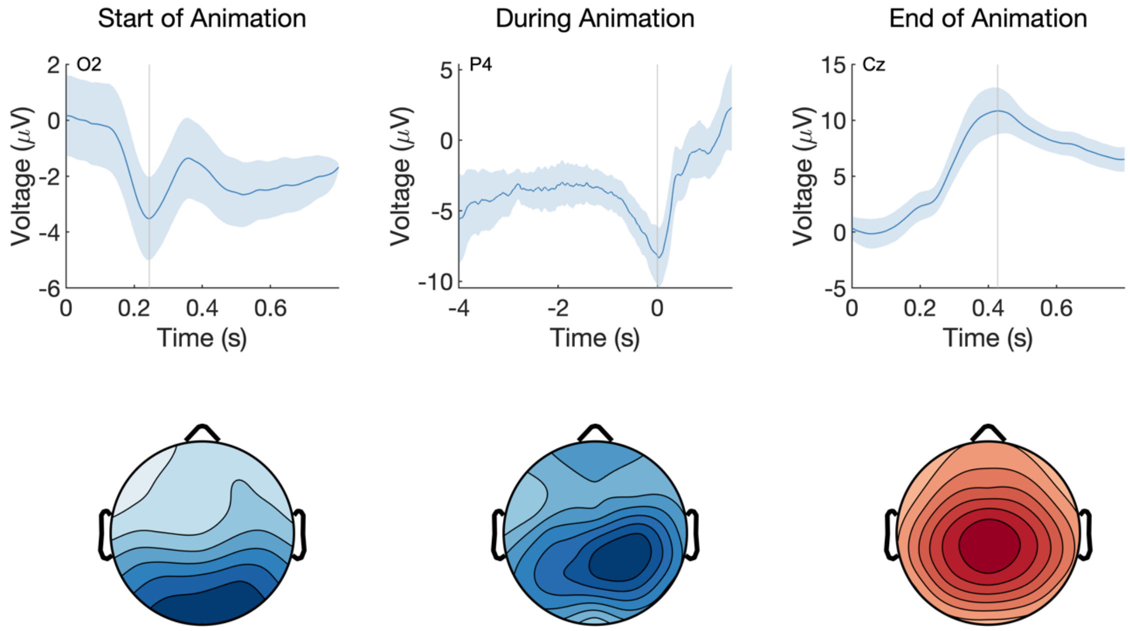

Supplementary Figure S5. ERPs before, during, and after the animation of the bar indicating the outcome of a trial. The plotted electrodes show the maximum signal location. The shaded regions indicate 95% confidence intervals. The scalp topographies represent the voltage at each electrode at the sample indicated by the vertical grey line.
